# Supplementary material for: Transcriptional profiling reveals the transcription factor networks regulating the survival of striatal neurons
Source: Cell Death Dis. 2021 Mar 12;12(3):262. doi: 10.1038/s41419-021-03552-8 (PMC7955055; doi:10.1038/s41419-021-03552-8)
Supplement: Supplementary file 1 — Supplemental Figure and Table Legends [file 41419_2021_3552_MOESM1_ESM.docx]

**Supplementary Figure Legends**

**Supplementary Figure 1. Pre-processing of RNA-Seq data**

**A** Correlation analysis of experiments between 5 time-points.

**B** Pearson correlation coefficient and Euclidean distance are averaged between two adjacent time points.

**C** Number of genes at each time-point after quality control.

**Supplementary Figure 2. DEG enrichment analysis between two adjacent time points.**

**A** DEG biological process was enriched between two adjacent time points. Some signiﬁcant GO terms are displayed.

**B** DEG KEGG pathway was enriched between two adjacent time points. Some signiﬁcant GO terms are displayed.

**Supplementary Figure 3.** **The expression profiles of PEGs between two adjacent time points and enrichment analysis.**

**A** Expression patterns of PEGs between two adjacent time points. The number of genes and TFs in each module are shown on the right.

**B** PEG biological processes were enriched between two adjacent time points. Some signiﬁcant GO terms are displayed.

**Supplementary Figure 4.** **Construction of the co-expression network.**

**A** Analysis of network topology for different soft‐threshold powers. The left panel shows the impact of soft‐threshold power on the scale‐free topology fit index; the right panel displays the impact of soft‐threshold power on the mean connectivity.

**B** Network heatmap plot of the co-expression genes and functional modules. The progressively saturated red colours suggested the higher overlap among those functional modules.

**Supplementary Figure 5. The enrichment analysis of each co-expression module.**

**A** The different co-expression module was enriched in biological processes. Some signiﬁcant GO terms are displayed.

**B** Different co-expression modules were enriched in KEGG pathways. Some signiﬁcant GO terms are displayed.

**Supplementary Figure 6.** **The workflow of machine learning and network annotation.**

**A** The workflow of machine learning.

**B** Degree distribution in different predicted interaction networks. The distributions followed a descending linear pattern. The Y and X axes represent the number of nodes and number of neighbors, respectively.

**C** Circular bar chart showing overlaps of the TFs with homologous genes from The Brain Atlas database.

**D** Circular bar chart showing the overlap of the TFs with nervous system–associated disease genes from the DisGeNET database.

**Supplementary Figure 7.** **Properties of TF networks.**

The functional significance distribution of predicted TF networks and random TF networks.

**Supplementary Figure 8.** **The** **quantification regions of the striatum.**

The schema chart of quantification regions in this study (modified from Atlas of the

Developing Mouse Brain).

**Supplementary Table Legends**

Table S1 Genes identified at 5 time-points.

Table S2 DEGs identified and enriched between two adjacent time points.

Table S3 Comparations of striatal development and HD.

Table S4 PEGs identified and enriched between two adjacent time points.

Table S5 Co-expression analysis of CEGs and module enrichment.

Table S6 Training sets for machine learning.

Table S7 Prediction of functional TFs networks based on machine learning.

Table S8 DEGs identified and enriched in Six3-CKO mice.

Table S9 DEGs identified and enriched in Meis2-CKO mice.

Table S10 Custom TFs and neuroassociated diseases databases.

Table S11 Information of HD patients.
