# Supplementary figures and images for: Transcriptional profiling reveals the transcription factor networks regulating the survival of striatal neurons
Source: Cell Death Dis. 2021 Mar 12;12(3):262. doi: 10.1038/s41419-021-03552-8 (PMC7955055; doi:10.1038/s41419-021-03552-8)

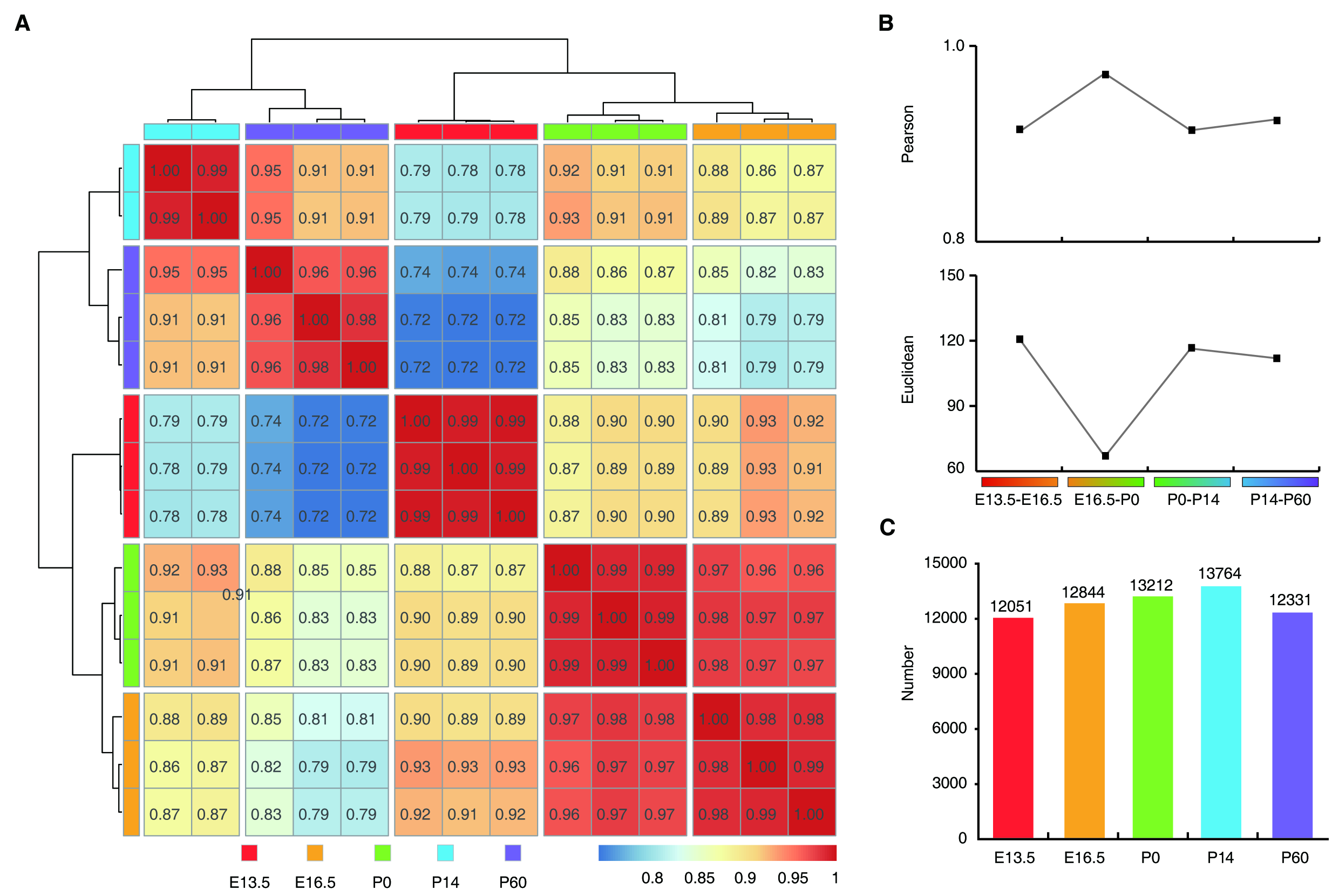

Supplement: Supplementary file 2 — Figure S1 [file 41419_2021_3552_MOESM2_ESM.tif]

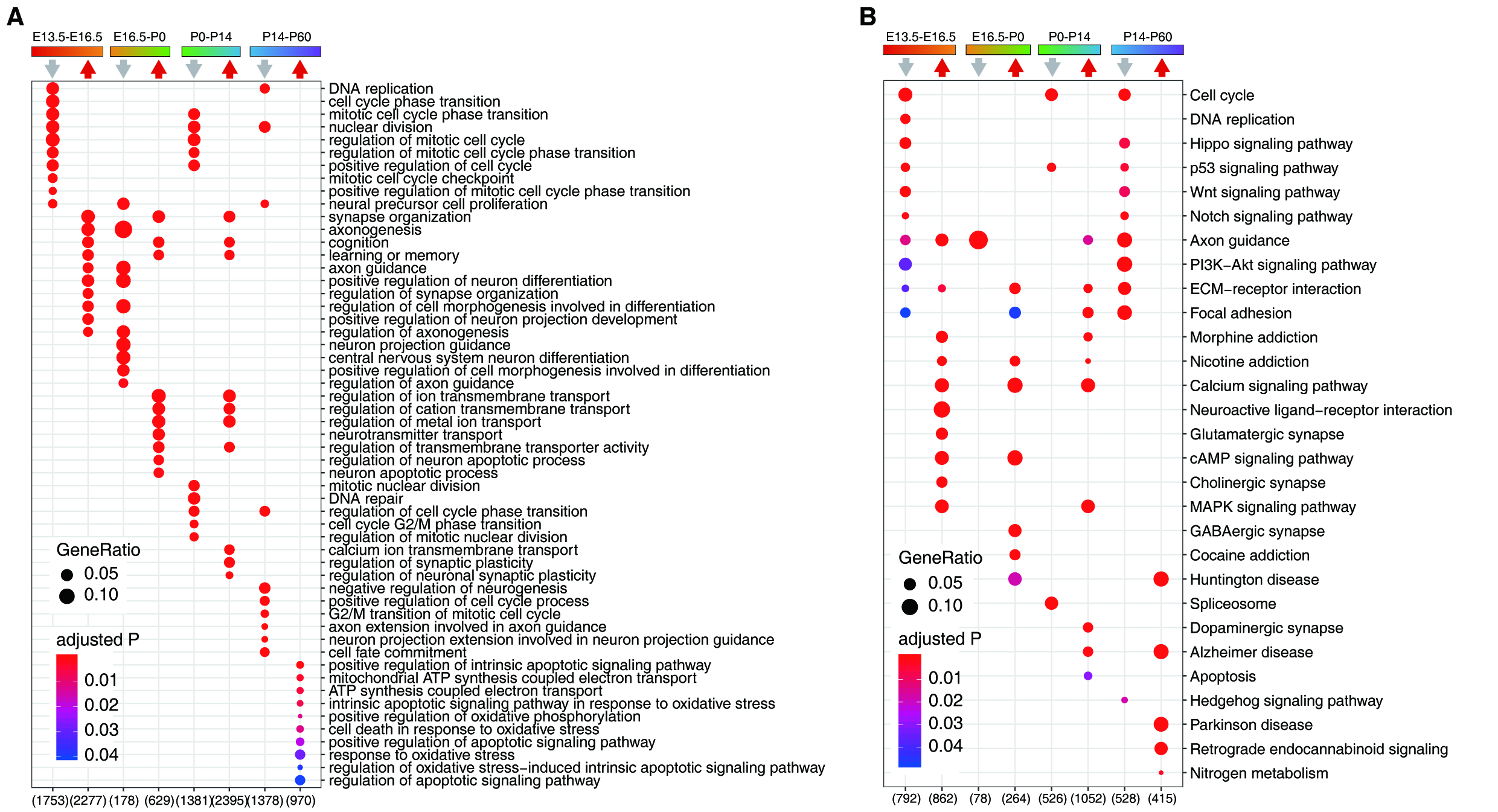

Supplement: Supplementary file 3 — Figure S2 [file 41419_2021_3552_MOESM3_ESM.tif]

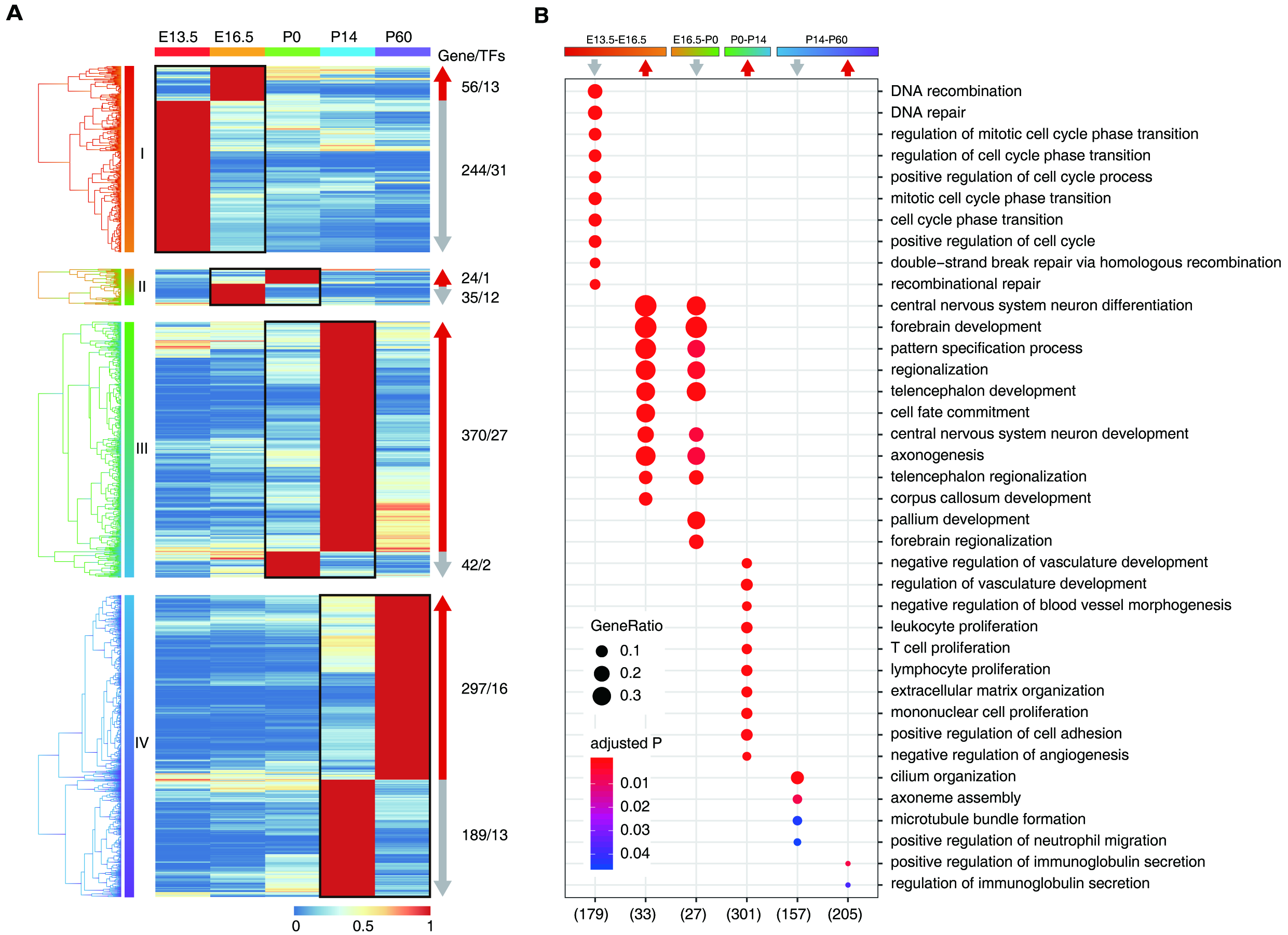

Supplement: Supplementary file 4 — Figure S3 [file 41419_2021_3552_MOESM4_ESM.tif]

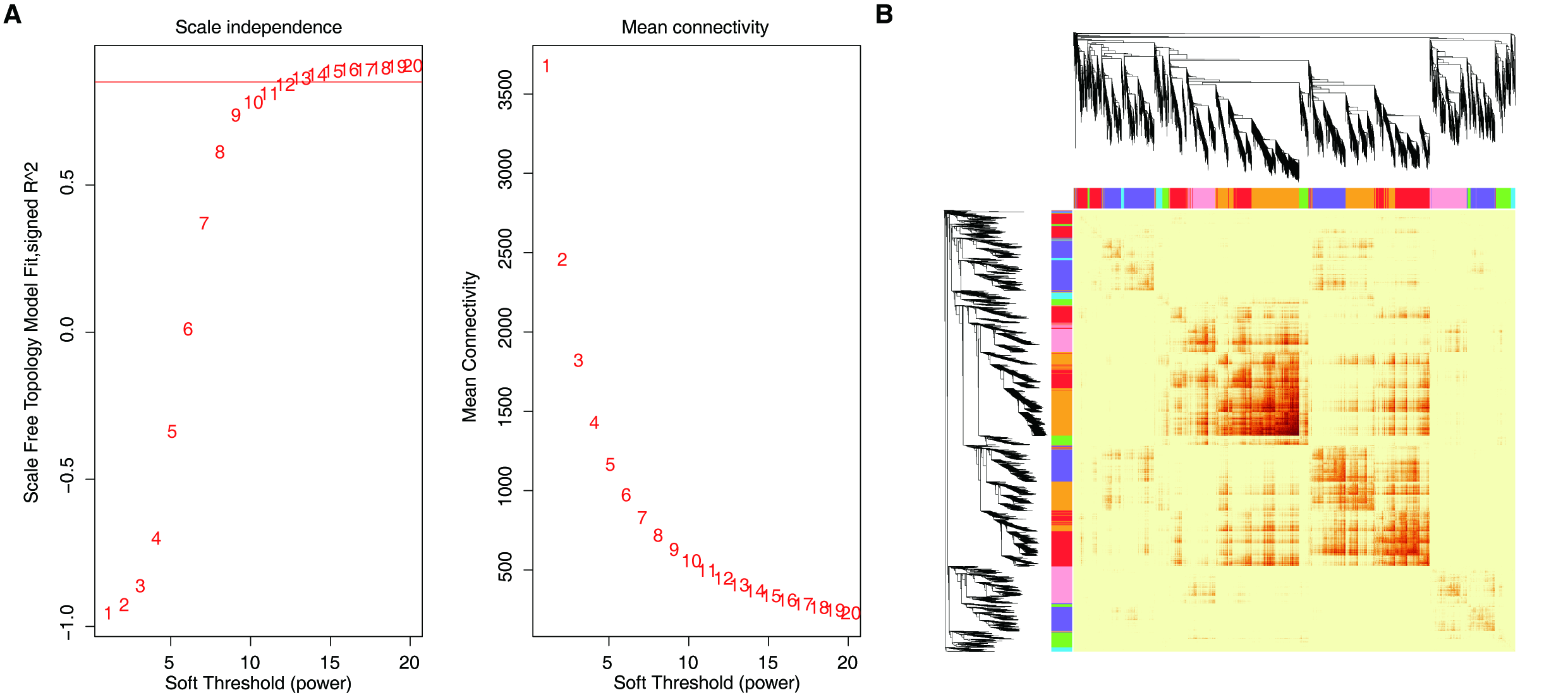

Supplement: Supplementary file 5 — Figure S4 [file 41419_2021_3552_MOESM5_ESM.tif]

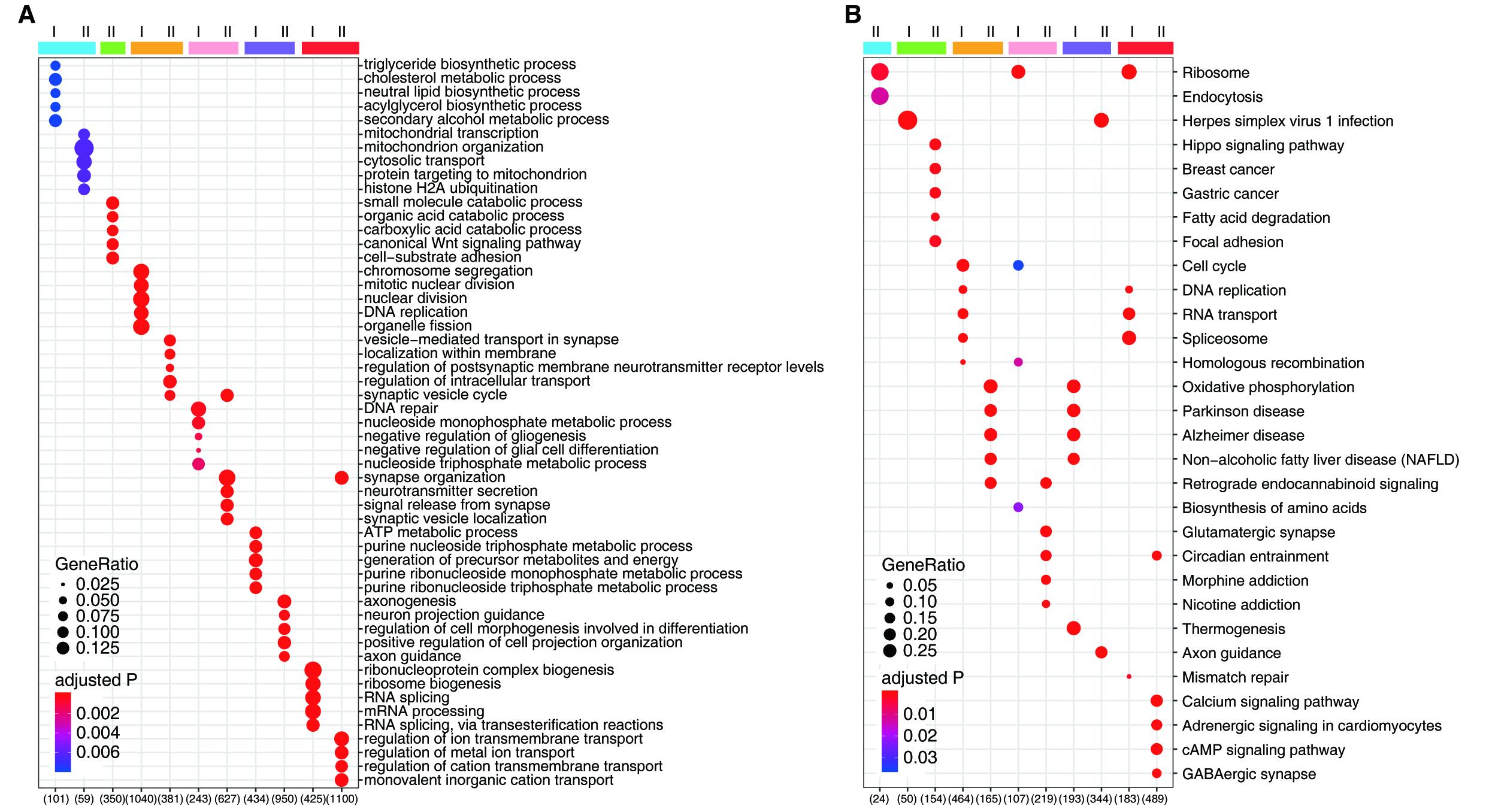

Supplement: Supplementary file 6 — Figure S5 [file 41419_2021_3552_MOESM6_ESM.tif]

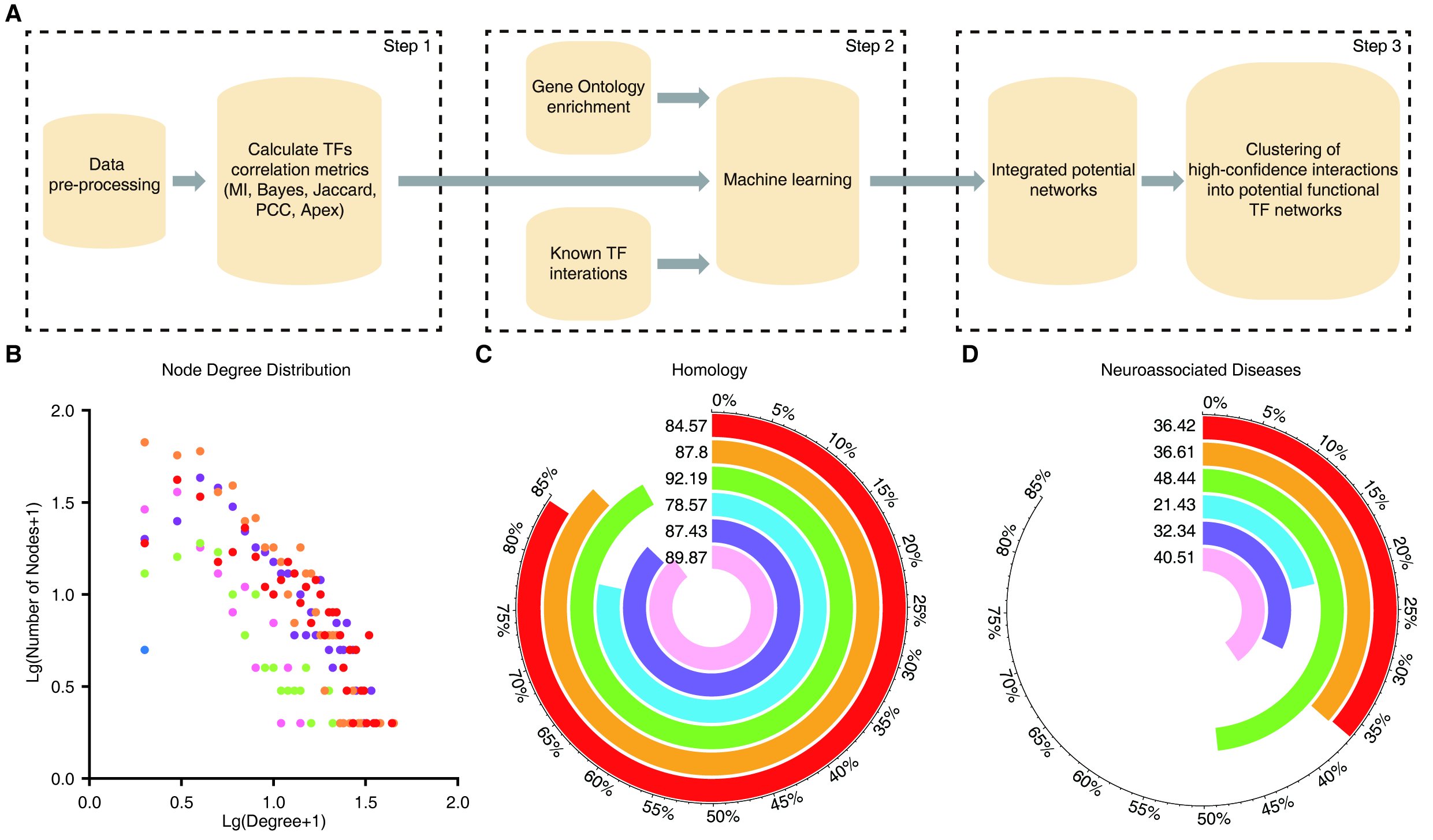

Supplement: Supplementary file 7 — Figure S6 [file 41419_2021_3552_MOESM7_ESM.tif]

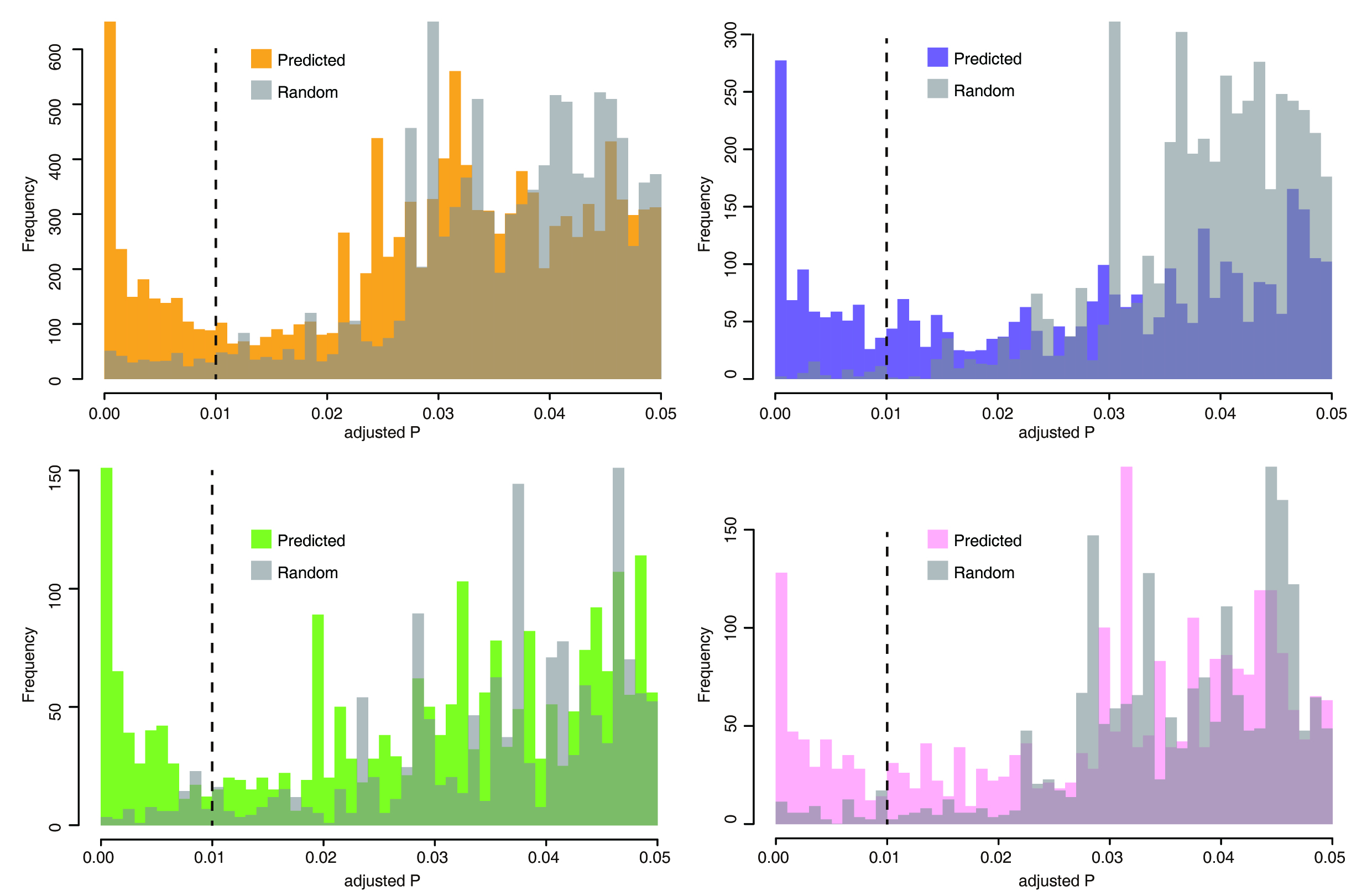

Supplement: Supplementary file 8 — Figure S7 [file 41419_2021_3552_MOESM8_ESM.tif]

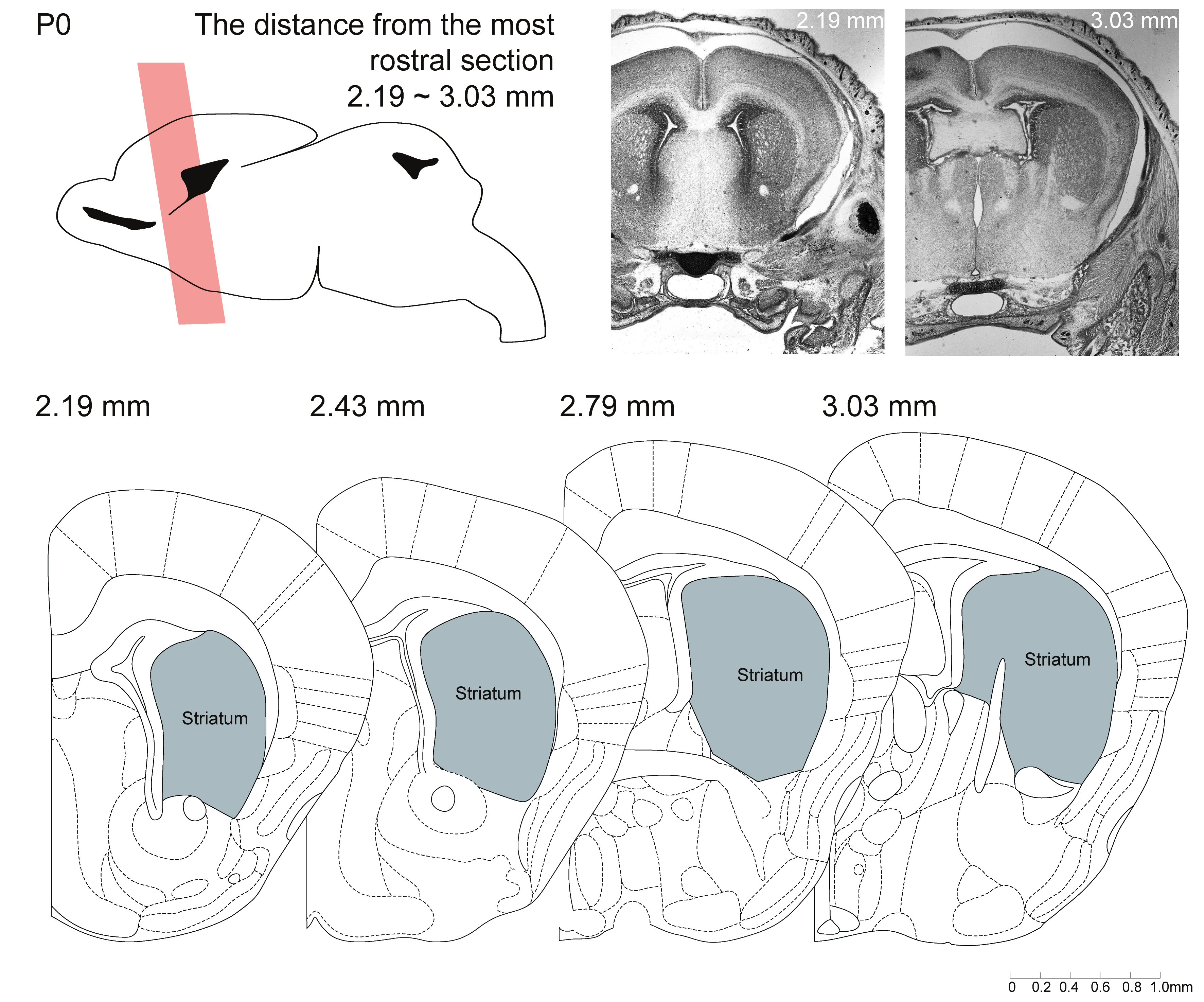

Supplement: Supplementary file 9 — Figure S8 [file 41419_2021_3552_MOESM9_ESM.tif]
